# Recent Preclinical Evidence on Phytocannabinoids in Neurodegenerative Disorders: A Focus on Parkinson’s and Alzheimer’s Disease

**DOI:** 10.3390/ph18060890

**Published:** 2025-06-13

**Authors:** Nicoleta-Mirela Blebea, Ciprian Pușcașu, Gabriel Hancu, Alina Mihaela Stăniguț, Cornel Chiriță

**Affiliations:** 1 Department of Pharmacotherapy, Faculty of Pharmacy, “Ovidius” University of Constanța, 900470 Constanța, Romania; nicoleta.blebea@gmail.com; 2 Department of Pharmacology and Clinical Pharmacy, Faculty of Pharmacy, “Carol Davila” University of Medicine and Pharmacy, Traian Vuia 6, 020956 Bucharest, Romania; cornel.chirita@umfcd.ro; 3 Department of Pharmaceutical Chemistry, Faculty of Pharmacy, “George Emil Palade” University of Medicine, Pharmacy, Science and Technology of Târgu Mureș, 540142 Târgu Mureș, Romania; gabriel.hancu@umfst.ro; 4 Department of Clinical Medical Disciplines, Faculty of Medicine, “Ovidius” University of Constanța, 900470 Constanta, Romania; alina.stanigut@365.univ-ovidius.ro; 5 Nephrology Department, Constanta County Emergency Clinical Hospital, 145 Tomis Street, 900591 Constanta, Romania

**Keywords:** endocannabinoid system, cannabinoids, cannabidiol, tetrahydrocannabinol, Alzheimer’s disease, Parkinson’s disease, neurodegenerative disorders

## Abstract

The endocannabinoid system (ECS) is a vital biological network essential for maintaining homeostasis and supporting various physiological functions. It comprises cannabinoid receptors, endogenous lipid-based ligands, known as endocannabinoids, as well as metabolic enzymes and associated proteins responsible for regulating their levels within tissues. The ECS plays a central role in modulating processes involving the central nervous system (CNS). Recent studies have highlighted its antioxidant, anti-inflammatory, and neuroprotective properties. The therapeutic potential of cannabinoids, particularly phytocannabinoids derived from plants, has attracted significant attention in medical and pharmaceutical research. This interest has grown in parallel with the increasing availability of cannabinoid-based food supplements on the pharmaceutical market. Given the complexity of the ECS and its broad range of interactions, the discovery of this system has spurred extensive investigations into the use of cannabinoids for various health conditions. In this review, we examine recent preclinical evidence supporting the use of phytocannabinoids in the context of neurodegenerative diseases, particularly in Alzheimer’s disease and Parkinson’s disease. Targeting the ECS through phytocannabinoid-based pharmacological modulation offers a promising therapeutic strategy for these neurological disorders. Among these compounds, cannabidiol has emerged as a key focus of research due to its multifaceted effects and favorable safety profile. Nonetheless, continued investigation is necessary to clarify its mechanisms of action, and to develop effective, evidence-based clinical applications.

## 1. Introduction

Phytocannabinoids are a group of oxygenated aromatic hydrocarbon metabolites derived from the *Cannabis* plant, typically characterized by a 21-carbon atomic structure [1]. They are currently classified into 11 major chemical classes, including cannabidiol (CBD), tetrahydrocannabinol (THC), cannabichromene (CBC), cannabielsoin (CBE), cannabigerol (CBG), cannabicyclol (CBL), cannabinol (CBN), cannabinodiol (CBND), cannabitriol (CBT), (−)-Δ8-trans-tetrahydrocannabinol (Δ8-THC), and other structurally related phytocannabinoid derivatives. These classes differ in terms of their oxidation states, cyclization patterns, and side chain modifications, contributing to their distinct pharmacological profiles [2].

**Figure 1:**
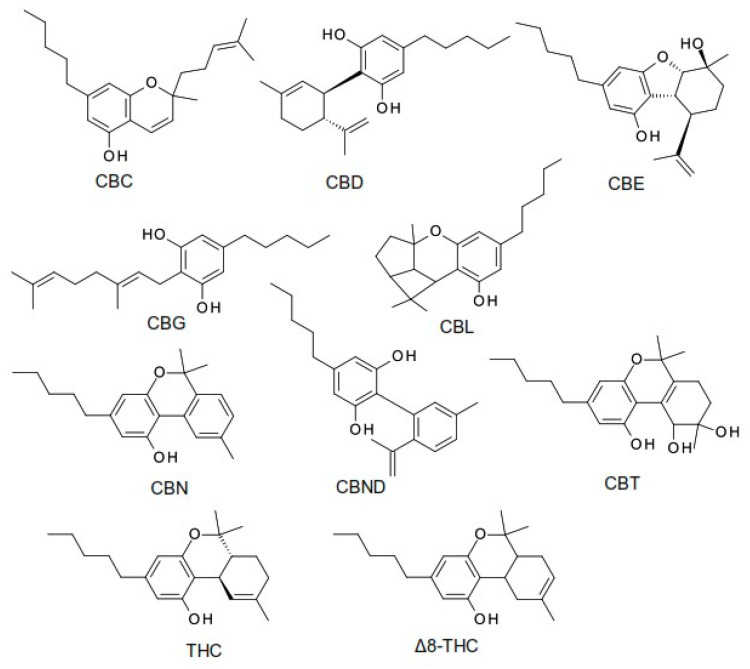
Chemical structures of the most common phytocannabinoids [1,2].

The chemical structures of the main phytocannabinoid derivatives are shown in Figure 1.

The variability in phytocannabinoid concentrations across different *Cannabis* chemotypes has important implications for the formulation and administration of medical cannabis products [1]. While the ECS primarily functions through endogenous ligands (endocannabinoids, or eCBs), numerous plant-derived cannabinoids have been identified that also interact with this system. Initial discoveries have focused on compounds isolated from *Cannabis sativa*, which contains over 150 phytocannabinoids, along with other bioactive terpenophenolic constituents [3,4].

Interestingly, cannabinoid-like compounds have also been identified in various non-cannabis plants and foods,. including the following: truffles, which contain anandamide (acting on the CB_1_ and CB_2_ receptors); echinacea, which produces alkamides (CB_2_); maca, with macamides (CB_1_); kava, which contains yangonin (CB_1_); and black pepper and cloves, which are dietary sources of β-caryophyllene, a selective CB_2_ receptor agonist with potential anti-inflammatory and neuroprotective effects. Additionally, other phytochemicals interact with the receptors linked to the ECS, such as capsaicin from hot peppers (transient receptor potential vanilloid 1, TRPV1), piperine from black pepper (TRPV1), gingerol and zingerone from ginger (TRPV1), and cocoa-derived compounds like N-oleoylethanolamine and N-linoleoylethanolamine, which inhibit fatty acid amide hydrolase (FAAH) [3,4].

The most recognized phytocannabinoids are Δ^9^-tetrahydrocannabinol (THC) and cannabidiol (CBD), which are also the most prevalent compounds found in *Cannabis* strains. THC mainly produces its effects by functioning as a partial agonist of the CB_1_ receptor, which is primarily located in the CNS. This interaction is responsible for the well-documented psychoactive and, in some cases, adverse effects associated with THC consumption. By contrast, CBD exhibits a broad and complex range of pharmacological actions. Unlike THC, CBD has a low affinity for the CB_1_ and CB_2_ receptors, but can influence the ECS through indirect pathways. These include inhibiting the reuptake and degradation of endogenous cannabinoids, activating the transient receptor potential vanilloid 1 (TRPV1) channel, and interacting with the G protein-coupled receptor 55 (GPR55). Furthermore, CBD has been shown to enhance the activity of the serotonin 5-HT1A receptors, which may underlie its anxiolytic and antidepressant-like effects [5].

In addition to its well-documented anti-inflammatory and antioxidant properties, CBD also demonstrates anxiolytic, antidepressant, antipsychotic, and anticonvulsant effects. Unlike THC, which functions as a partial agonist for both the CB_1_ and CB_2_ receptors—producing psychoactive and analgesic effects mainly via CB_1_ activation—CBD exhibits minimal affinity for the orthosteric binding sites of these receptors. Instead, it may mitigate some of THC’s actions by acting as a negative allosteric modulator of the CB_1_ receptor [6,7]. Beyond the canonical endocannabinoid receptors, CBD interacts with a range of non-cannabinoid targets, including the 5-HT1A receptors, transient receptor potential channels, and nuclear receptors [8,9]. Notably, CBD has demonstrated a favorable safety and tolerability profile in humans, with a low potential for abuse. This has contributed to a relaxation of legal restrictions in many countries, increasing its availability and popularity for therapeutic use [10].

However, the surge in consumer interest has outpaced scientific validation and regulatory oversight. As a result, the market has become saturated with unregulated CBD products, particularly dietary supplements sold online. Consumers often lack the tools or resources to verify the chemical composition and quality of these products, making them vulnerable to purchasing substandard or mislabeled items [11,12]. With the growing recognition of CBD’s therapeutic potential, particularly in conditions characterized by oxidative stress and inflammation—such as cardiovascular, neurodegenerative, oncological, and metabolic disorders—ensuring the consistency, purity, and cannabinoid profile of CBD products is increasingly important for both efficacy and safety [13,14].

Currently, the U.S. Food and Drug Administration (FDA) has approved one cannabis-derived pharmaceutical product: Epidiolex^®^, an oral solution containing CBD [15]. In addition, three synthetic cannabis-related medications have been approved: Sativex^®^ (nabiximols), an oromucosal spray combining CBD and THC in a 2.5:2.7 mg ratio; Cesamet™, which contains nabilone—a synthetic analogue of THC; and Marinol^®^, which contains dronabinol, another synthetic form of THC [15].

Epidiolex^®^ is approved for the treatment of seizures associated with Lennox–Gastaut syndrome and Dravet syndrome in patients aged two years and older [16,17]. Sativex^®^ has been authorized in several countries—including the United Kingdom (2010), Germany and Denmark (2011), and France (2013)—for the management of spasticity, neuropathic pain, and other symptoms associated with multiple sclerosis. Cesamet™ is mainly prescribed to alleviate chemotherapy-induced nausea and vomiting, while Marinol^®^ is indicated for the treatment of anorexia related to weight loss in patients with AIDS, as well as for the nausea and vomiting caused by chemotherapy [15].

In parallel with pharmaceutical developments, CBD has gained widespread popularity as an ingredient in over-the-counter products, including dietary supplements, essential oils, skincare formulations, functional foods, and complementary medicines. Its wide-ranging pharmacological profile makes it a promising candidate for adjunctive therapy in a variety of conditions, such as epilepsy, anxiety, neuropathic pain, and cancer [18,19,20]. Additionally, the appetite-stimulating effects of THC—commonly referred to as hyperphagia—have been linked to the activity of endogenous cannabinoids within the ECS [18].

Phytocannabinoids are lipophilic compounds that are rapidly absorbed in the body. Most of the available pharmacokinetic data pertain to CBD and THC. The pharmacokinetic profiles of these compounds can vary considerably among individuals, influenced by factors such as dosage, formulation, frequency of use (acute versus chronic), and route of administration. Inhalation methods, such as smoking or vaporizing cannabis, result in higher blood concentrations of cannabinoids, faster onset of action, and greater bioavailability compared to oral administration [8].

With increasing global life expectancy, neurodegenerative diseases are emerging as a significant socio-economic challenge. In 2014, approximately 1.6% of the population was affected by Alzheimer’s disease and related dementia [21,22]. In the United States, between 2016 and 2017, an estimated 4.7 to 6 million people were living with neurodegenerative conditions, resulting in 272,644 deaths [23]. By 2020, the combined economic burden of Alzheimer’s disease (AD), amyotrophic lateral sclerosis (ALS), Parkinson’s disease (PD), and spinal muscular atrophy had reached $655 billion [21,22]. According to the World Health Organization (WHO), neurodegenerative diseases are projected to become the second leading cause of death worldwide by 2040 [24].

Despite differences in pathophysiology, clinical manifestations, and age-specific prevalence, AD, PD, ALS, and Huntington’s disease (HD) share several critical features. Notably, none of these disorders currently have effective long-term treatments, as available therapies often lose efficacy over time and are associated with considerable side effects. Furthermore, the progressive nature of these conditions imposes a substantial socio-economic burden, necessitating prolonged palliative care that significantly impacts both patients and caregivers [25].

In recent decades, the ECS has garnered increasing attention as a key modulator of various physiological and pathological processes [26]. Both preclinical and clinical studies have shown alterations in components of the ECS in animal models and human patients with neurodegenerative diseases [27]. Moreover, recent research in disease models has highlighted the potential of ECS modulation as a therapeutic strategy, with promising results in improving neurodegenerative outcomes in animals [28,29]. The intricate nature of the ECS provides important insights into the neurodegenerative and neuroinflammatory processes involved in disorders such as AD, PD, HD, and ALS. This deeper understanding of disease pathophysiology aids in the identification of new therapeutic targets [30]. Given the growing interest in the therapeutic role of phytocannabinoids, and the urgent need for novel treatments in neurodegenerative diseases, this narrative review aims to provide a comprehensive and up-to-date synthesis of recent preclinical studies, with a particular focus on the mechanistic insights and experimental findings related to AD and PD. By emphasizing the most relevant data from the past five years, this review offers a novel perspective on the neuroprotective potential of phytocannabinoids, and identifies future research directions in this evolving field. Thus, our article contributes a current, focused, and mechanistically rich narrative on the phytocannabinoid research in neurodegeneration, particularly for AD and PD, filling a critical gap in the recent literature.

## 2. Endocannabinoid System

The identification of the endogenous cannabinoid system at the end of the 20th century renewed scientific interest in the therapeutic potential of cannabinoids. The ECS is a ubiquitous signaling network present in all vertebrates, with essential regulatory functions throughout the body [31,32]. As a neuromodulatory system, the ECS plays a critical role in maintaining and modulating various physiological processes involving the CNS [33], as well as influencing endocrine activity, reproductive function, cardiovascular health, and overall homeostasis. It acts as a chemical interface between the body and the mind, making it a vital biological component for survival, and a growing target in pharmacotherapy.

The ECS is composed of cannabinoid receptors (CB_1_ and CB_2_), endogenous lipid ligands (endocannabinoids, or eCBs), metabolic enzymes, and additional proteins and cofactors necessary for optimal system functioning [32]. Its effects are observable at multiple levels—from cellular mechanisms to complex behavioral outcomes, underscoring their significance in human health.

From a chemical perspective, the ECS is a lipidergic system involving a wide array of substances related to lipid metabolism. These include precursor molecules, metabolic enzymes, active mediators, and final metabolic products. In addition to classical endocannabinoids, the ECS also encompasses allosteric modulators, such as lipoxins and resolvins, as well as peptide-based ligands, like hemopressin derivatives. The system is further modulated by exogenous compounds, including phytocannabinoids (e.g., THC and CBD) and synthetic cannabinoids (e.g., WIN55,212-2). The primary enzymes responsible for endocannabinoid metabolism are FAAH and monoacylglycerol lipase (MAGL), alongside specific membrane transporters that regulate eCB distribution and activity [31].

## 3. Cannabinoid Receptors

Initially, the physiological effects of cannabinoids (CBs) were thought to result from nonspecific interactions with cell membranes. However, research in the late 1980s using animal models led to the identification of specific cannabinoid receptors. The first such receptor, CB_1_ (short for “cannabinoid binding” receptor), was described in 1988 by the team of William Devane and Allyn Howlett in the United States [33]. A few years later, in 1993, the CB_2_ receptor was identified by Sean Munro and his colleagues in the United Kingdom [34].

Currently, two subtypes of cannabinoid receptors, called CB_1_ and CB_2_, are known to mediate most of the physiological effects of cannabinoids. These receptors differ in their function, localization, and the distinct physiological responses they elicit upon activation. Both the CB_1_ and CB_2_ receptors are members of the G protein-coupled receptor (GPCR) superfamily—transmembrane proteins that play a key role in cellular signal transduction [35].

**Figure 2:**
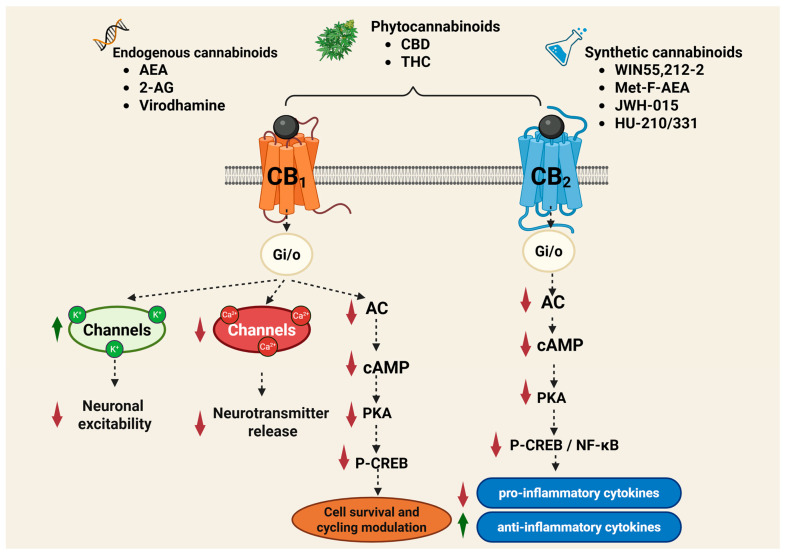
Cannabinoid Receptor Signaling Pathways ( 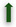 activation/increase; 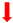 inhibition/decrease; 2-AG—2-arachidonoylglycerol; AC—adenylate cyclase; AEA—anandamide (N-arachidonoylethanolamine); cAMP—cyclic adenosine monophosphate; CB_1_—cannabinoid receptor type 1; CB_2_—cannabinoid receptor type 2; CBD—cannabidiol; P-CREB—phosphorylated cAMP response element-binding protein; Gi/o—inhibitory G protein (Gi/o subtype); NF-κB—nuclear factor kappa-light-chain-enhancer of activated B cells; PKA—protein kinase A; THC—tetrahydrocannabinol. The figure was created using BioRender (web application, accessed May 2025; BioRender Inc., Toronto, ON, Canada; www.biorender.com).

Cannabinoid receptors are activated by three major classes of ligands: endogenous cannabinoids (endocannabinoids or eCBs), phytocannabinoids (e.g., THC, CBD), and synthetic cannabinoids (e.g., WIN55,212-2; 2-methyl-2′-F-anandamide [Met-F-AEA]; JWH-015; HU-210; HU-331) (Figure 2). Cannabinoids bind to these receptors in a reversible and stereoselective manner, and their affinity for each receptor determines their specific biological effects. Compounds with higher selectivity for a particular receptor subtype are often preferred in medical applications to reduce side effects and increase therapeutic efficacy [36].

Due to their high lipophilicity, both natural and synthetic cannabinoids exhibit strong membrane permeability and efficient interaction with the lipid-rich environment of the CB_1_ and CB_2_ receptors. For example, THC has a log *p* value of approximately 6.4, CBD around 6.3, and synthetic cannabinoids, such as HU-210, can exceed a log P of 7. These values reflect their strong partitioning into lipid membranes, which not only facilitate receptor binding but contribute to their prolonged retention in fatty tissues and central nervous system structures. The lipophilic nature of these compounds is a key determinant of their pharmacokinetics, tissue distribution, and affinity for intracellular or mitochondrial receptor sites [5,8,10].

Cannabinoids exert their effects by binding to and modulating the activity of the CB_1_ and CB_2_ receptors. These receptors are critically involved in a wide range of physiological functions, including neuronal plasticity, neuroregeneration, nociception and pain signaling, cognitive and emotional regulation, metabolic processes and appetite control, immune responses and inflammation, sleep regulation, motor coordination, and even tumor suppression [35,36].

CB_1_ receptors are predominantly expressed in the CNS, particularly in the brain regions involved in motor control, emotional regulation, motivation, and homeostasis [35]. They are also found in the peripheral nervous system (PNS) and various peripheral tissues, including adipose tissue, liver, pancreas, skeletal muscle, the gastrointestinal tract, cardiovascular system, and reproductive organs such as the ovaries, testicles, and endometrium [36]. Notably, elevated expression of the CB_1_ and CB_2_ receptors has been observed also in cancer cells, which may represent a therapeutic target for limiting tumor growth and propagation [37].

CB_2_ receptors are primarily located in immune cells and tissues, including the spleen, thymus, bone marrow, tonsils, and circulating immune cells such as B lymphocytes, T lymphocytes (CD4+ and CD8+), macrophages, neutrophils, and natural killer (NK) cells [38,39]. Although originally considered a peripheral receptor due to its limited expression under physiological conditions, CB_2_ has also been identified in microglial cells within the CNS, where it plays an active role in neuroimmune signaling. Its cellular signaling mechanisms are largely like those of CB_1_ [38,39,40,41].

Stimulation of the CB_1_ and CB_2_ receptors elicits both central and peripheral effects. CB_1_ is implicated in the regulation of cognition, memory, appetite, sleep–wake cycles, mood, thermogenesis, and pain perception. CB_2_, on the other hand, is central to immune modulation and inflammatory control, influencing immune cell migration, cytokine release, and antigen presentation in both the CNS and PNS. Pharmacological agents targeting these receptors can enhance, inhibit, or fine-tune their activity, providing promising avenues for therapeutic interventions. The ability to modulate cannabinoid receptor activity and influence cellular functions across nearly all organ systems opens new possibilities for the treatment of a wide range of pathological conditions [42].

### 3.1. Distribution of Cannabinoid Receptors

#### 3.1.1. CB_1_ Receptors

CB_1_ receptors were initially identified in the brain, where their distribution has been extensively mapped using techniques such as in situ hybridization and immunohistochemistry. These studies reveal that CB_1_ receptors are predominantly localized on presynaptic terminals [43]. The highest concentrations are found in the olfactory bulb, hippocampus, basal ganglia, and cerebellum. By contrast, lower levels are present in the thalamus and the ventral horn of the spinal cord. Moderate expression is observed in the cerebral cortex, septum, amygdala, hypothalamus, and various regions of the brainstem [43,44].

Researchers do not exclude the presence of CB_1_ receptors at postsynaptic sites and, to a lesser extent, in astrocytes, oligodendrocytes, and microglia, where they are known to modulate synaptic transmission [45,46,47]. In these cortical regions, CB_1_ receptors are primarily expressed by two major neuronal subpopulations: GABAergic interneurons, which display high levels of receptor expression, and glutamatergic neurons, which exhibit comparatively lower levels. These two populations represent opposing forces in regulating brain excitability, with GABAergic neurons providing inhibitory and glutamatergic neurons providing excitatory signaling [48].

CB_1_ receptors are also present in neurons of the dorsal raphe nucleus and the locus coeruleus, which are the brain’s principal sources of serotonin (5-HT) and norepinephrine, respectively. Therefore, direct or indirect modulation of monoaminergic, GABAergic, or glutamatergic activity may underlie the psychotropic and non-psychotropic effects of CB_1_ receptor activation.

In the PNS, CB_1_ receptors are found in high amounts in nerve endings [48], including the trigeminal ganglion and primary sensory neurons, particularly in the regions involved in nociception along afferent fibers [49]. Through both neural and non-neural pathways, CB_1_ receptors modulate gastrointestinal motility, gastric acid secretion, neurotransmitter and hormone release, and intestinal epithelial permeability. Consequently, CB_1_ receptors influence appetite and energy balance, and play a regulatory role in the metabolism [50].

Under normal physiological conditions, CB_1_ receptor expression in the liver is low. However, under pathological conditions, expression increases. Similarly, CB_1_ receptors are upregulated in the cardiovascular system, where they contribute to disease progression and cardiac dysfunction. Activation of the CB_1_ receptors in cardiomyocytes, vascular endothelial cells, and smooth muscle has been linked to oxidative stress, inflammation, and fibrosis [51]. In addition, CB_1_ receptors have been detected in adipose tissue, skeletal muscle, skin, eyes, the reproductive system, and various cancer cells [52]. Numerous studies have also reported a predominant intracellular localization of CB_1_ receptors, particularly in mitochondria, where their activity has been associated with mitochondrial function and energy regulation [43].

The endocannabinoid system undergoes significant region-specific alterations across the lifespan, with notable changes in CB_1_ receptor expression and signaling. In aged rodents, several studies have reported a decline in the CB_1_ receptor density, particularly in the cerebellum and cerebral cortex, with more moderate reductions observed in the limbic and hypothalamic regions [53,54]. In humans, however, the findings are more heterogeneous and, in some cases, contrast with those reported in rodents. Postmortem analyses have similarly shown age-related reductions in CB_1_ receptor binding, especially in the cortical areas [55]. An in vitro study comparing brain tissue from children (aged 3 months to 8 years) and adults (22 to 73 years) found significantly higher CB_1_ receptor density in adults, particularly in the frontal cortex, hippocampus, caudate putamen, globus pallidus, and cerebellum [56]. Supporting this, another PET study reported increased CB_1_ receptor binding in aged females across several brain regions, including the basal ganglia and limbic system, whereas no significant age-related differences were observed in males [57]. By contrast, a PET imaging study in healthy male subjects identified only a slight age-associated decline in CB_1_ receptor binding, with statistical significance limited to the globus pallidus [58]. These contrasting findings between animal and human studies may reflect species-specific differences, methodological variability, or sex-related hormonal influences. Collectively, they highlight the complexity of the endocannabinoid system regulation across the human lifespan.

Interestingly, a significant increase in the CB_1_ receptor density has been observed in the dorsolateral prefrontal cortex (PFC) of individuals who died by suicide with a history of depression, suggesting possible hyperactivity of the ECS in this population. By contrast, other studies have reported a downregulation of ECS activity in depression, with reduced CB_1_ receptor density in glial cells in the gray matter and decreased serum levels of 2-AG in patients with major depressive disorder. Additionally, patients with minor depression have shown significantly increased serum levels of 15(S)-hydroxy-arachidonylethanolamide (HAEA). Two clinical trials reported correlations between elevated eCB levels and hypertension in depressed women. Additionally, a link was observed between intense physical activity, increased anandamide (AEA) levels, and BDNF concentrations. These findings suggest a possible interrelationship between eCBs, depression, cardiovascular risk, and peripheral BDNF levels [59].

In conclusion, CB_1_ receptors are found in neural tissues, particularly in the basal ganglia, cerebellum, hippocampus, and frontal cortex. They are present to a lesser extent in the hypothalamus and spinal cord, and are absent from the cardiorespiratory nuclei in the brainstem, which may explain the impossibility of lethal intoxication via CB_1_ receptor overactivation [59].

CB_1_ receptors have also been identified in the PNS and in various tissues and organs, including the prostate, adrenal glands, bone marrow, heart, lungs, thymus, tonsils, and spleen, as well as in immune cells, adipocytes, hepatocytes, and musculoskeletal tissues. Furthermore, they are present in the lungs, liver, and kidneys. Their expression in chondrocytes and osteocytes, along with emerging evidence of their presence in fibroblasts, has increased research interest in their role in rheumatic diseases [60].

#### 3.1.2. CB_2_ Receptors

Shortly after the discovery of the CB_1_ receptor, another receptor—CB_2_—was identified in macrophages and the spleen [34]. CB_2_ is primarily expressed in the immune system, where it plays a key role in regulating immune function [36]. Subsequent studies confirmed a predominant presence of CB_2_ receptors in immune cells and a moderate expression in various peripheral tissues, including the cardiovascular and gastrointestinal systems, liver, adipose tissue, bone, and reproductive organs [43].

Initially, unlike the CB_1_ receptor, CB_2_ was not thought to be present in the CNS, and was therefore referred to as the “peripheral cannabinoid receptor”, with its activity considered mostly immune-related. This early interpretation stemmed from a lack of detectable CB_2_ expression in the CNS using available methods. However, this understanding has significantly evolved with newer research revealing a more nuanced role of CB_2_ in the brain. Its dual action—within both the immune system and the CNS—underscores its potential as a promising therapeutic target. Engagement of the CB_2_ receptors may facilitate neuroprotection, reduce neuroinflammatory responses, and offer new avenues for the treatment of psychiatric disorders [61].

CB_2_ receptors, although best known for their presence in immune and inflammatory cells, are also activated by endocannabinoids like AEA and 2-AG. While these receptors are predominantly found in immune-related tissues, they have also been identified in glial cells and, to a lesser extent, in neurons located in brain regions such as the cerebral cortex, hippocampus, amygdala, hypothalamus, and cerebellum. Their established function involves modulating inflammatory responses and pain perception. However, recent studies have indicated that CB_2_ receptors may also play a role in emotional regulation. The detection of endocannabinoid system components in brain areas linked to emotional processing—such as the prefrontal cortex, hippocampus, amygdala, and hypothalamus—strengthens the rationale for exploring cannabinoid-based therapies in mood and affective disorders [61].

Mackie demonstrated the role of CB_2_ receptors in neurological functions such as nociception, drug addiction, and neuroinflammation [59]. Studies have also shown intracellular localization of CB_2_ receptors in pyramidal neurons of the PFC, where they modulate neuronal excitability through regulation of chloride channels activated by calcium ions. Similarly, in human osteosarcoma epithelial cells, intracellular CB_2_ receptors regulate calcium levels more rapidly and effectively than those located at the cell surface [43].

In conclusion, CB_2_ receptors are abundantly expressed in immune-related tissues, including the palatine tonsils, thymus, spleen, bone marrow, lymphocytes, NK cells, monocytes/macrophages, neutrophils, and both B and T lymphocytes (CD8+, CD4+) [38,39], as well as in hematopoietic cells [62]. Additionally, they are found in skeletal muscle and CNS-resident immune cells, such as microglia, cerebro-microvascular endothelial cells, and astrocytes. Their expression is notably upregulated during inflammatory responses. The study by Onaivi et al. was one of the first studies to show that CB_2_ receptors and their gene transcripts are more extensively distributed throughout the CNS under normal physiological conditions than was previously recognized [63].

Activation of the CB_2_ receptor leads to a complex immune regulatory response. CB_2_ receptor activation also inhibits adenylyl cyclase (AC) and mitogen-activated protein kinases system (MAPKs), and can increase intracellular calcium levels through phospholipase C (PC)-mediated pathways [64].

### 3.2. Physiological and Pathological Roles of CB_1_ Receptors

Considering the distribution of CB_1_ receptors in the human body, they perform a multitude of physiological roles. The ECS is involved in various aspects of central neural activity and a range of disorders, including anxiety, depression, schizophrenia, stroke, multiple sclerosis, neurodegenerative disorders, epilepsy, and addiction [43]. The CB_1_ receptor plays a role in various physiological and pathological processes within the peripheral nervous system (PNS) and peripheral tissues. It is involved in the regulation of pain, energy metabolism, cardiovascular and reproductive functions, inflammation, glaucoma, cancer, liver disorders, and musculoskeletal conditions [43].

Studies have shown that CB_1_ receptors inhibit the release of GABA and glutamate from presynaptic endings, giving them the ability to modulate neurotransmission—a core mechanism underlying CB_1_ receptor-mediated neuroprotection against excitotoxicity, a pathological process common in many neurological disorders such as epilepsy and neurodegenerative diseases [43]. Chiarlone et al. demonstrated that only CB_1_ receptors situated on glutamatergic terminals in mice contribute to the neuroprotective effect against excitotoxicity [65].

Studies have also revealed a direct interaction between the CB_1_ and NMDA receptors (NMDARs), which enables CB_1_ to negatively regulate NMDAR activity and protect neuronal cells from excitotoxicity [66,67].

The upregulation of CB_1_ receptors and the increased ECS activity observed in the basal ganglia of experimental PD models may reflect either a compensatory response to dopaminergic neuron loss in the substantia nigra or a pathological mechanism contributing to disease progression [68]. A decrease in the ECS activity has also been reported in PD patients, in whom FAAH inhibitors and CB_1_ receptor antagonists improve motor symptoms. In animal models, CB_1_ receptor activation has been shown to prevent amyloid β-induced neurotoxicity in several cell models [69,70]. Researchers have also reported that CB_1_ receptor activation is beneficial in animal models of AD, improving memory deficits and cognitive impairments [71].

In 1993, Glass et al. first reported the benefits of CB_1_ receptors in HD, noting a decrease in CB_1_ receptor expression in the substantia nigra of patients with HD [72]. Subsequent research has revealed a progressive loss of CB_1_ receptors as an early event in HD patients, occurring before overt neurodegeneration. This decrease was also observed at the mRNA level and in CB_1_ receptor immunoreactivity across various transgenic HD mouse models [73].

Zoppi et al. demonstrated that a delayed loss of CB_1_ receptors in R6/1 HD transgenic mice is associated with the delayed onset of motor symptoms, suggesting a slower disease progression. Conversely, in R6/2 HD transgenic mice, deletion of the CB_1_ receptors led to worsening motor performance [74]. Selective upregulation of CB_1_ receptor expression in the adrenal medulla has been shown to promote neuronal survival without improving motor function in R6/2 Huntington’s disease (HD) mice. By contrast, Aso et al. [71] found that CB_1_ receptor activation reduces motor impairments and striatal atrophy in transgenic mouse models [70]. Another study using HD cell models showed that CB_1_ receptor activation protects striatal cells from excitotoxic damage by upregulating BDNF expression through the PI3K/Akt signaling pathway [75].

The literature data also highlight the antiepileptic effects mediated by CB_1_ receptor activation, demonstrating beneficial outcomes in patients with seizures and in animal models of epilepsy. Activation of CB_1_ by AEA has been shown to inhibit electroshock-induced seizures in rats [43]. In patients with acute-phase epilepsy, a decreased CB_1_ receptor density is observed in the hippocampal tissue, particularly within the dentate gyrus, while in the chronic phase of the disease, CB_1_ receptor upregulation has been reported [76,77].

In cases of low CB_1_ expression in the hypothalamus, cannabinoids exert appetite-stimulating effects. Endocannabinoid levels increase in the rat hypothalamus during fasting and return to baseline after feeding [78]. Additionally, CB_1_ receptor activation inhibits GABAergic neurons, producing a hyperphagic, but not orexigenic effect [79].

The CB_1_ receptor has the highest preponderance of all G-protein receptors in the brain, compared to μ opioid receptors, D_2_ dopamine receptors (D_2_R) or similar ones [6]. CB_1_ is a presynaptic localized receptor with the highest density in the hippocampus, cerebellum, basal ganglia and cortex, and in immune cells. The intricate tapestry of encephalic regions where the CB_1_ receptor is consistently observed weaves through several pivotal areas. Notably, one finds this receptor nestled within the substantia nigra and globus pallidus, as well as the dentate gyrus and the expansive hippocampus [80]. The cerebral cortex, particularly vibrant in its frontal domain, along with the anterior cingulate and the occipital architectural zones—specifically layers I and VI—are also significant sites. Delving deeper, the striatum, comprising the caudate nucleus and putamen, emerges as a crucial player. The cerebellum and amygdala contribute their own unique dynamics, while the thalamus and hypothalamus stand as central hubs of processing. Interestingly, this receptor does not limit itself to the brain; it extends its expression into the sensory neurons of the posterior ganglion roots and the periapeductal gray matter, highlighting a remarkable breadth of influence. The CB_1_ receptor is found in varying, smaller amounts, in peripheral neurons or in the heart, lungs, liver, kidneys, testicles, prostate, ovaries, uterus, gastrointestinal tract, bones, bone marrow, and thymus [18]. In neuronal cells, the receptor is mainly presynaptic in the axonal endings, corresponding to the GABAergic, glutamatergic, cholinergic, and dopaminergic neurons in the somatodendritic portion being minimally detectable [81].

Thus, considering the recent preclinical evidence regarding ECS activation and its effects on promoting neurogenesis, it is plausible that ECS activation also contributes to antidepressant effects through mechanisms like those triggered by conventional antidepressants, particularly those influencing synaptic plasticity.

### 3.3. Physiological and Pathological Roles of CB_2_ Receptors

Multiple studies have confirmed the analgesic effects of cannabinoids in various pain models, including chemical and mechanical pain, as well as neuropathic, inflammatory, and cancer-associated pain. CB_2_ selective agonists have demonstrated considerable effectiveness in preclinical models of neuropathic pain, while the increase in the number of clinical trials has confirmed the potential of the CB in terms of benefits for patients with chronic pain and chronic inflammatory conditions (arthritis) [82]. The ECS is involved in regulating nociception; eCBs play a role in controlling inflammatory and neuropathic pain, with numerous studies demonstrating the effect of CBD in modulating chronic pain. The drug Sativex^®^, which has been approved in the United States, is used to treat various MS-related symptoms, including chronic pain [83]. Although CBD has minimal affinity for the CB_1_ and CB_2_ receptors, the study by Laprairie et al. suggested that it acts as an allosteric modulator at concentrations below 1 μM, and functions as an indirect antagonist of cannabinoid receptors, with the capacity to enhance the effects of THC [9].

### 3.4. Mechanisms of Action

The activation of the CB_1_ receptor by means of the G protein produces inhibition of Ca^2+^ channels type N-, P/Q and L-, activation of K^+^ channels (GIRK), including type I M and I A, stimulation of the MAPKs, focal adhesion kinase (FAK), phosphatidyl-inosotol-3-kinase (PI3K), stimulation of phospholipase A and C, and the production of nitric oxide (NO) (Figure 2). The CB_1_ receptor inhibits adenylate cyclase (AC) but can activate the formation of cyclic adenosine monophosphate (cAMP) at over-added coupling with the Gs protein, when, at the same time, the usual attachment to a Gi/o protein, known as heterodimerization, is activated [84,85].

The activity of the CB_1_ receptor controls the transmission of nerve impulses, synaptic plasticity and remodeling of neuronal circuits, cell proliferation and regeneration, which translate into effects correlated with nociception, motricity and locomotion, psychoemotional and cognitivemnesic profile, metabolic and immunological activity rate, control of inflammation and ischemia, but also in other effects with therapeutic applications [86].

The cellular signal mechanism of the CB_2_ receptor is largely like the central CB_1_ receptor. Thus, when activating the CB_2_ receptor, the release of cytokines T-helper 1 is inhibited, such as interleukin 2 (IL-2), interferon gamma (IFN-γ), TNFα; at the same time, the release of cytokines Th2, type IL-4, IL-5, IL-10, having other modulation actions in inflammatory and immune processes, increases (Figure 1). The effects of the CB_2_ receptor, intrinsic or conditioned by other factors, such as CB_1_ receptor coactivation or endocannabinoid involvement, are related to the tissue and anti-inflammatory, regenerative and antioxidant protective aspect [87]. The low expression of the CB_2_ receptor under normal physiological conditions, explains why it was first considered a peripheral cannabinoid receptor. Its presence in the CNS and PNS is relatively limited, with the CB_2_ receptor playing an active role in neurological activities, such as nociception, drug addiction and neuroinflammation [40,41].

Activation of the CB_1_ and CB_2_ receptors produces both central and peripheral effects. CB_1_ receptors are involved in regulating cognition, memory, appetite, sleep–wake cycles, emotional responses, thermogenesis, and nociception. By contrast, the CB_2_ receptors play a role in modulating immune responses and inflammatory processes by influencing immune cell migration, cytokine release, and antigen presentation to the CNS. The action of the drugs can potentiate, protect, or inhibit these well-defined relationships. The beneficial modulation of cannabinoid receptor activity and the modification of cellular functions in almost all tissues and organs will allow the implementation of new therapeutic possibilities in all the pathologies evoked [86].

## 4. Phytocannabinoids in the Treatment of Neurodegenerative Disorders

### 4.1. Effects of Phytocannabinoids in Alzheimer’s Disease

Alzheimer’s disease (AD) is among the most severe disorders of the CNS, characterized by progressive and irreversible neuronal degeneration. Affecting over 50 million people worldwide, it presents primarily as cognitive decline, often accompanied by disturbances in behavior, language, motor function, mood, and overall daily functioning—factors that collectively impair quality of life [88,89,90,91]. The pathological features of AD are characterized by significant brain lesions caused by the accumulation of extracellular senile plaques, primarily composed of insoluble β-amyloid (Aβ) peptides. These peptides initiate a cascade of neurodegenerative processes that ultimately result in neuronal death.

Another defining feature of AD is the accumulation of tau proteins within neurons. Normally associated with microtubules, tau proteins become misfolded and hyperphosphorylated, leading to the formation of intracellular neurofibrillary tangles. These aggregates interfere with intracellular transport and contribute to cellular dysfunction and neurodegeneration [89,92,93,94].

Genetic mutations also play a key role in some cases of AD. Mutations in genes encoding the amyloid precursor protein (APP) and presenilins 1 and 2 (PSEN1 and PSEN2)—all of which are involved in the production of Aβ peptides—are directly linked to familial forms of AD. These inherited forms of the disease typically have an early onset and, although they account for only 1–5% of all cases, they are considered critical in understanding the disease’s underlying mechanisms [95].

In addition to genetic predisposition, a variety of non-genetic and environmental risk factors have been associated with increased susceptibility to AD. These include increasing age, exposure to environmental toxins and viral agents, traumatic brain injuries, cardiovascular disease, diabetes, and a sedentary lifestyle—all of which may contribute to the onset and progression of the disease [96,97,98].

Studies involving animal models and post-mortem brain tissue from AD patients have revealed notable alterations in the ECS. Several studies have reported a reduction in CB_1_ receptor expression in key brain regions, including the cortex and hippocampus, which play essential roles in memory and cognitive function [99,100]. By contrast, CB_2_ receptor expression is consistently found to be upregulated with AD, where its activation suppresses pro-inflammatory cytokine release, reduces microglial reactivity, and promotes a shift toward a more neuroprotective phenotype [101].

Furthermore, reduced plasma levels of the endocannabinoid 2-AG have been correlated with cognitive decline, suggesting a potentially protective role for 2-AG in AD pathophysiology [102]. Notably, the enzymes involved in 2-AG metabolism—diacylglycerol lipase (DAGL), responsible for its synthesis, and MAGL, responsible for its degradation—are elevated in the hippocampus of post-mortem AD brains, indicating dysregulated endocannabinoid signaling during advanced disease stages [103].

Several recent preclinical studies have explored the effects of phytocannabinoids on AD (Table 1). Coles et al. [104] examined behavioral and cognitive alterations in 12-month-old female APPxPS1 transgenic mice, a widely used model for AD. These mice exhibited hyperactivity, heightened anxiety, cognitive deficits—including delays in spatial and reversal learning—and impairments in object recognition, as well as abnormalities in sensorimotor gating. The animals were treated with intraperitoneal (i.p.) injections of CBD at a dose of 5 mg/kg daily for three weeks. Remarkably, the CBD treatment reversed object recognition deficits and significantly improved spatial learning performance. These findings suggest that a moderate-dose CBD regimen may offer selective therapeutic benefits for certain behavioral and cognitive impairments associated with AD. Additionally, the study highlights the potential value of lower-dose CBD approaches, which could reduce treatment costs and minimize side effects. In a separate study using a tauopathy-based animal model, daily administration of a higher CBD dose (100 mg/kg) resulted in the restoration of motor function, normalization of reduced body weight, and alleviation of anxiety-like behavior observed in transgenic mice. Notably, CBD also corrected social interaction deficits and reversed impairments in spatial reference memory, without producing significant adverse effects [105].

Chronic CBD treatment in APP/PS1 mice enhanced hippocampal immune responses and autophagy, as revealed by RNA-Seq (ribonucleic acid **sequencing**), immunohistochemistry, electron microscopy, and Western blot analyses. Immunohistochemistry also showed a reduction in Aβ plaques. These effects, coupled with down-regulated oxidative phosphorylation and TNF signaling, suggest improved mitochondrial functioning and reduced inflammation, highlighting CBD’s potential as a therapeutic agent for AD [106]. Conversely, in AβPPxPS1 transgenic mice, administration of 50 mg/kg of CBD effectively improved cognitive deficits—particularly social recognition memory and spatial learning—and moderately reduced insoluble Aβ_40_ levels in the hippocampus, but showed no significant impact on neuroinflammation, neurodegeneration, or PPAR-γ expression in the cortex [107]. However, despite the positive effects of CBD in transgenic AD mouse models, Watt et al. reported that chronic CBD treatment (50 mg/kg) did not improve behavioral changes in transgenic males, such as heightened anxiety and impaired motor functions. Interestingly, this study also highlighted that 4-month-old TAU58/2 transgenic males exhibited no deficits in social recognition memory, suggesting that motor impairments and anxiety-related changes at this age do not significantly affect social behavior [108].

Glucose hypometabolism is an early and consistent feature of AD. A recent study using an streptozotocin (STZ)-induced AD model examined the effects of CBD on brain glucose metabolism. Animals treated with CBD (i.p., one week) showed preserved memory in novel object recognition tests. By contrast, the control group displayed reduced 18 fluorodeoxyglucose ([18F]FDG) uptake, with hypometabolism detected in regions near the lateral ventricle, including the striatum, motor cortex, hippocampus, and thalamus. These deficits were absent in the CBD group, suggesting CBD may offer early neuroprotective benefits for AD [109].

Arnanz et al. explored the effects of a chronic ultra-low-dose treatment with CBD (0.273 mg/kg), THC (0.205 mg/kg), or a CBD–THC combination (0.273:0.205 mg/kg) in the 5xFAD mouse model of AD. Mice treated with THC alone displayed anxiogenic and depressant-like behaviors, which were absent in the other groups. Notably, only the combination treatment led to significant improvements in spatial memory. All cannabinoid-treated groups showed increased cortical levels of insoluble β-amyloid 1–42, but without corresponding changes in inflammatory markers at either the mRNA or protein level [110]. In an aluminum chloride-induced AD model in rats, exposure to aluminum chloride led to nitrosative stress, elevated total protein levels, and increased cell death associated with a rise in β-amyloid. Subchronic treatment with an oily *Cannabis* sp. extract in a 2:1 THC–CBD ratio produced notable therapeutic effects. Specifically, doses above 100 µL reduced plasma nitrite levels, while doses from 50 µL normalized plasma total protein levels and prevented cell death [111].

Another study evaluated whether acute doses of CBD and rivastigmine, administered alone or in combination, could counteract scopolamine-induced memory deficits in mice using the passive avoidance test. Memory impairments, which are linked to cholinergic dysfunction, common in neurodegenerative diseases like AD, were significantly reduced by either CBD (1 mg/kg) or rivastigmine (0.5 mg/kg). Notably, the combined treatment produced a greater improvement across memory acquisition, consolidation, and retrieval than either drug alone. These findings suggest that a CBD–rivastigmine polytherapy may offer a more effective strategy for managing cognitive disorders associated with cholinergic deficits [112].

Most research on *Cannabis* focuses on CBD and THC, yet the plant contains up to 150 phytocannabinoids, including their acidic precursors, cannabidiolic acid (CBDA) and tetrahydrocannabinolic acid (THCA). These compounds can cross the blood–brain barrier and have demonstrated anti-inflammatory, neuroprotective, and pain-relieving properties. In this regard, the effects of CBDA and THCA were investigated on AD-like pathology in Aβ_1_₋_42_-treated mice and primary neurons. The Aβ_1_₋_42_-treated mice showed increased hippocampal Aβ and p-tau accumulation, calcium dyshomeostasis, and cognitive impairments. However, treatment with CBDA and THCA significantly reduced the Aβ and p-tau levels, restored calcium homeostasis, and improved cognitive function. Additionally, CBDA and THCA enhanced CREB (cAMP response element-binding protein) phosphorylation, leading to increased expression of BDNF and its receptor, p-TrkB (phosphorylated tropomyosin receptor kinase B), both essential for synaptic plasticity and memory [113].

In addition to rodent models, several studies have used *C. elegans* to explore how CBD alleviates AD in vivo. Transgenic *C. elegans* models have been developed for a variety of conditions, including AD. This organism offers significant advantages over rodents, such as a shorter lifespan, lower costs, easier manipulation, and access to a wide range of multicolor reporter constructs. Moreover, approximately 38% of its genome is homologous to human genes, including those coding for amyloid precursor protein (APP) and tau, underscoring its value as a model organism [114,115,116]. When administered at 100 μM, CBD enhanced the neural glyoxalase pathway to detoxify methylglyoxal, directly scavenged reactive oxygen species (ROS) through its phenolic hydroxyl groups, and inhibited amyloid-β aggregation, thereby protecting neurons and extending the lifespan [114,115]. At 50 μM, CBD improved the pumping rate and reduced mitochondrial ROS [116].

### 4.2. Effects of Phytocannabinoids in Parkinson’s Disease

Parkinson’s disease (PD) is the second most common neurodegenerative disorder after AD, affecting around 1–2% of individuals over the age of 70. With life expectancy on the rise, the number of PD cases is projected to increase substantially, potentially reaching up to 9 million cases in the next decade [117,118]. The disease is characterized mainly by the progressive degeneration of dopaminergic neurons in the substantia nigra, driven by factors such as chronic inflammation, oxidative stress, genetic susceptibility, and abnormal protein aggregation. Clinically, PD manifests through a combination of motor and non-motor symptoms, including muscle rigidity, bradykinesia, tremor, postural instability, sleep disturbances, fatigue, impaired coordination, memory decline, vocal changes, and cognitive dysfunction [89,119].

In patients with PD, an upregulation of CB_2_ receptor expression has been observed, whereas the CB_1_ receptor levels appear to remain stable, suggesting a potentially important role for CB_2_ in the pathogenesis of the disease [119]. Both endogenous and exogenous cannabinoids are known to modulate basal ganglia circuits. Specifically, activation of presynaptic CB_1_ receptors decreases GABA release from striatal terminals projecting onto dopaminergic neurons. Additionally, stimulation of both the CB_1_ and CB_2_ receptors has been shown to suppress glutamate release from cortical inputs to the striatum. Although CB_1_ receptors are not expressed on dopaminergic neurons themselves, their regulatory effect on neurotransmitter release indirectly impacts dopamine synthesis within the dorsal striatum [120].

Currently, preclinical studies are increasingly focused on exploring the potential of phytocannabinoids to alleviate PD-related symptoms (Table 2). In this context, Giuliano et al. examined the impact of chronic CBD administration (10 mg/kg, i.p.) in the 6-hydroxydopamine (6-OHDA) model of PD, focusing on neurodegeneration, neuroinflammation, motor deficits, and underlying mechanisms. The CBD-treated animals exhibited reduced nigrostriatal degeneration, decreased neuroinflammation, and improved motor function. Notably, CBD’s effects appeared to be mediated mainly through astrocytic TRPV1 activation, which triggered a neuroprotective response via increased ciliary neurotrophic factor (CNTF) production [121].

In addition to these effects, CBD exhibits notable antiparkinsonian and neuromodulatory properties, which are likely mediated through interactions with multiple molecular targets, including the recently identified GPR55 receptor. Given that GPR55 is expressed in key motor regions, such as the external globus pallidus (GPe) and the striatum, a separate study evaluated the impact of intrapallidal administration of CBD and a selective GPR55 antagonist (CID16020046) in hemiparkinsonian rats. The CBD treatment significantly reduced motor asymmetry, as evidenced by decreased amphetamine-induced turning, and markedly improved fine motor skills. Improvements were particularly evident in contralateral forelimb performance during the staircase test, as well as in pronation, grasp, and supination tasks. Moreover, the CBD treatment led to a reduction in GAD-67 expression within the striatum and ipsilateral GPe, indicating decreased GABAergic overactivation. Collectively, these findings suggest that CBD’s therapeutic effects in this PD model may be largely attributed to its modulation of GPR55 activity, resulting in the alleviation of motor impairments [122].

Pain affects approximately 60% of the individuals with PD, manifesting as nociceptive, neuropathic, or other forms, often accompanied by lower pain thresholds and the development of allodynia. The dopaminergic deficit within the basal ganglia is thought to play a key role in these altered pain perceptions. Despite increased analgesic usage among PD patients (33%) compared to the general population (20%), pain remains frequently underrecognized and insufficiently managed [123]. Recent studies have examined how 6-hydroxydopamine (6-OHDA)-induced Parkinsonism alters nociceptive responses in murine models, and evaluated the modulatory effects of CBD on pain sensitivity. To elucidate the mechanisms underlying CBD’s analgesic action, researchers co-administered a FAAH inhibitor—known to elevate endogenous AEA levels—and selectively targeted anandamide-sensitive receptors, such as CB_1_, CB_2_, and TRPV1.

The results demonstrated that 6-OHDA-induced PD mice significantly lowered both thermal and mechanical pain thresholds, leading to hyperalgesia and allodynia. Notably, both acute and chronic CBD administration effectively mitigated these symptoms. Furthermore, subeffective doses of either the FAAH inhibitor or a TRPV1 antagonist potentiated CBD’s antinociceptive effects, whereas co-administration of inverse agonists at the CB_1_ and CB_2_ receptors abolished them. These findings suggest that CBD may help preserve nociceptive thresholds in PD through mechanisms involving CB_1_ and TRPV1 receptor activity, likely mediated by increased AEA levels [123].

Vivanco-Estela et al. further investigated the local effects of CBD on orofacial pain by administering varying concentrations directly into the masseter muscle in a PD animal model, using both male and female rats. Across all stages of the estrous cycle, female rats consistently exhibited greater allodynia and hyperalgesia compared to males. The estrous cycle—comprising the proestrus, estrus, metestrus, and diestrus phases—is driven by cyclical ovarian hormone fluctuations, which are known to influence pain sensitivity and must be carefully considered in experimental design. Among the phases, estrus was selected for detailed analysis due to its relatively stable hormonal profile. In both sexes, 6-OHDA lesions reduced mechanical and inflammatory pain thresholds, with more pronounced effects observed in females. Importantly, local CBD administration effectively reversed orofacial hyperalgesia and allodynia in both sexes. Females, however, responded to even the lowest CBD dose in terms of allodynia, while males exhibited a more substantial reduction in hyperalgesia in the formalin test. These findings underscore sex-dependent differences in CBD responsiveness, suggesting that hemiparkinsonian males and estrus-phase females may require individualized dosing strategies for optimal pain management [124].

In addition to CBD, THC has also been studied for its potential therapeutic effects in PD using 6-hydroxydopamine (6-OHDA) animal models. The findings from these studies demonstrated that THC alleviated motor deficits, including contralateral turning, catalepsy, and freezing behavior, while also improving motor coordination and balance. Moreover, THC improved cognitive performance, as demonstrated by enhanced spatial learning and memory in both the Morris water maze and novel object recognition tests. These beneficial effects were attributed to alterations in both the dopaminergic and the ECS. The THC treatment led to an upregulation of hippocampal dopamine D1 receptors (D1R) and selectively modulated CB_1_ and CB_2_ receptor expression, restoring their levels in 6-OHDA-lesioned rats. Additionally, THC increased the expression of postsynaptic density protein 95 (PSD-95), a synaptic scaffolding protein implicated in dopamine-related motor control. This upregulation is believed to support NMDA receptor-dependent signaling while reducing pathological D1–NMDA receptor interactions. Together, these findings suggest that THC exerts its motor and cognitive effects through a combination of dopaminergic and non-dopaminergic pathways, offering potential therapeutic value in PD management [125,126].

Beyond CBD and THC, other cannabis-derived compounds have also been investigated as potential therapeutic agents in experimental models of PD employing 6-hydroxydopamine (6-OHDA). One such compound is VCE-004.8, a 3-hydroxyquinone derivative of CBD that functions as an agonist at both CB2 and PPAR-γ. The neuroprotective and anti-inflammatory properties of VCE-004.8 were assessed in this context, with results demonstrating a significant attenuation of tyrosine hydroxylase (TH)-positive neuronal loss in the substantia nigra, indicating robust protection of dopaminergic neurons. Furthermore, VCE-004.8 elicited a marked reduction in neuroinflammatory markers, including a complete suppression of both astrogliosis and microgliosis. These findings suggest that its anti-inflammatory activity is closely associated with the preservation of neuronal integrity. Although the compound did not produce significant effects in the pole test, motor performance assessed via the cylinder rearing test showed substantial improvement. VCE-004.8 effectively reduced hemiparesis typically observed in 6-OHDA-lesioned mice, indicating enhanced motor function [127].

Another widely used animal model of PD, based on 1-methyl-4-phenyl-1,2,3,6-tetrahydropyridine (MPTP)-induced dopaminergic neurotoxicity, has also demonstrated favorable outcomes following phytocannabinoid treatment. In this model, the administration of CBD at a dose of 100 mg/kg significantly improved cognitive performance and enhanced spontaneous locomotor activity. A biochemical analysis revealed that the CBD treatment elevated the levels of 5-HT, dopamine (DA), and the anti-inflammatory cytokine interleukin-10 (IL-10), while concurrently reducing pro-inflammatory markers, including TNF-α, interleukin-1 beta (IL-1β), and interleukin-6 (IL-6). A histopathological examination further showed increased expression of tyrosine hydroxylase (TH), a critical enzyme in dopamine biosynthesis, suggesting preserved dopaminergic function. At the molecular level, CBD promoted neuronal survival by upregulating the anti-apoptotic protein B-cell lymphoma 2 (Bcl-2) and downregulating pro-apoptotic factors such as Bcl-2-associated X protein (Bax) and Caspase-3. Additionally, CBD inhibited activation of the NLRP3 inflammasome pathway—comprising the nucleotide-binding oligomerization domain (NOD), leucine-rich repeat (LRR), and pyrin domain-containing protein 3—along with its downstream effectors caspase-1 and IL-1β, a signaling axis central to neuroinflammation [128].

CBD significantly improved motor deficits and preserved the substantia nigra in a PD transgenic mouse model. It also modulated the gut–brain metabolic axis, affecting key metabolic pathways including fatty acid biosynthesis, arginine synthesis and metabolism, butanoate (ketone body) metabolism, β-alanine metabolism, and pantothenate/CoA biosynthesis. CBD’s neuroprotective effects on the midbrain contributed to the attenuation of PD symptoms, leading to improved motor performance. This therapeutic impact may be partially mediated by metabolic interactions between the gut and the brain, suggesting that CBD modulates energy production and essential substance biosynthesis in relation to PD pathogenesis [129].

The antioxidant and CB_2_ receptor agonist Δ^9^-tetrahydrocannabivarin (Δ^9^-THCV) was also investigated for its potential in PD. In a genetic model of dopaminergic deficiency (Pitx3ak mutant mice), Δ^9^-THCV demonstrated anti-dyskinetic properties. Administered i.p. at 2 mg/kg for two weeks, Δ^9^-THCV effectively delayed the onset and reduced the severity of L-DOPA-induced dyskinesia. When co-administered with L-DOPA from the first injection, it significantly postponed the appearance of abnormal involuntary movements and mitigated excessive motor activity. Furthermore, Δ^9^-THCV reduced the expression of the FosB protein and phospho-acetylated histone H3 (pAcH3) in the basal ganglia—markers commonly elevated in L-DOPA-induced dyskinesia. Beyond its preventive effects, Δ^9^-THCV also effectively lessened the severity of dyskinetic symptoms when administered for three consecutive days after dyskinesia had already manifested [130].

**Table 2:** Preclinical studies evaluating the effects of phytocannabinoids in PD.

Animal Model	Tested substance	Results	Reference
6-OHDA model Sprague–Dawley rats	CBD 10 mg/kg bw, i.p. 28 weeks	Enhanced motor activity through the activation of CNTF and astrocytic TRPV1 signaling.	[121]
6-OHDA model C57⁄BL6 mice	CBD 10, 30, and 100 mg/kg bw i.p. single treatment	Reduced the hyperalgesia and allodynia caused by 6-OHDA.	[123]
6-OHDA model Wistar rats	CBD 10, 50, 100 µ g/µL, i.m. single treatment	Reduced orofacial allodynia and hyperalgesia induced by the 6-OHDA lesion. Females (especially in the estrus phase) were more sensitive to the lowest dose for allodynia. Males showed a greater reduction in hyperalgesia in the formalin test.	[124]
6-OHDA model Wistar rats	CBD 10 nM, intrapallidal injection 3 days	Lowered amphetamine-induced motor asymmetry. Improved fine motor skills (staircase test, pronation, grasp, and supination). Decreased GAD-67 expression in the striatum and ipsilateral GPe.	[122]
6-OHDA model Sprague–Dawley rats	THC 5.1 mg/kg bw, i.p. 26 days	Significantly reduced apomorphine-induced contralateral rotations, beam travel time, beam freeze time, and catalepsy time. Increased latency to fall in the rotarod test. Improved balance time, and elevated protein levels of PSD-95 and dopamine receptor D1.	[125]
6-OHDA model Wistar rats	THC 0.85 mg/mL, i.p. 28 days	Improved spatial learning and memory. Increased hippocampal D1 mRNA levels. Decreased CB_1_ mRNA levels. Increased CB_2_ mRNA levels.	[126]
6-OHDA C57BL/6 mice	VCE-004.8 20 mg/kg bw, orally 2 weeks	Partially prevented the loss of TH-positive neurons in the substantia nigra. Nearly eliminated astroglial and microglial reactivity. Improved motor performance in the cylinder rearing test.	[127]
MPTP model SPF C57BL/6 mice	CBD 100 mg/kg bw, orally 14 days	Enhanced cognitive function. Increased spontaneous locomotion. Increased levels of DA, 5-HT, IL-10, and TH expression. Decreased levels of TNF-α, IL-1β, and IL-6. Upregulated Bcl-2. Downregulated Bax and Caspase-3.	[128]
Pitx3ak mutant mice that received repeated administration of L-DOPA	Δ^9^-THCV 2 mg/kg bw, i.p. 2 weeks	Delayed the onset and reduced the severity of dyskinesia. Attenuated already-established dyskinesia. Reduced AIMs and hyperactivity. Lowered levels of FosB protein and histone pAcH3 in the basal ganglia.	[130]
* aSyn * A53T transgenic mice	CBD 4.3 mg/kg i.p. 24 days	Significantly improved motor and postural coordination. Protected the substantia nigra. Modulated fatty acid biosynthesis, arginine metabolism, butanoate metabolism, β-alanine metabolism, and pantothenate/CoA biosynthesis.	[129]

Δ^9^-THCV—Δ^9^-tetrahydrocannabivarin; 6-OHDA—6-hydroxydopamine; aSyn A53T—alpha-synuclein A53T mutation; AIMs—abnormal involuntary movements; Bax—Bcl-2-associated X protein; Bcl-2—B-cell lymphoma; bw—body weight; CBD—cannabidiol; CB_1_—cannabinoid receptor 1; CB_2_—cannabinoid receptor 2; CNTF—ciliary neurotrophic factor; DA—dopamine; D1—dopamine receptor D1; GAD-67—glutamate decarboxylase 67; GPe—globus pallidus externus; i.m.—intramuscular; i.p.—intraperitoneal; IL-1β—interleukin-1 beta; IL-6—interleukin-6; IL-10—interleukin-10; L-DOPA—levodopa; MPTP—1-methyl-4-phenyl-1,2,3,6-tetrahydropyridine; mRNA—messenger ribonucleic acid; pAcH3—phospho-acetylated histone H3; PD—Parkinson’s Disease; PSD-95—postsynaptic density protein 95; SPF—specific pathogen-free; TH—tyrosine hydroxylase; THC—tetrahydrocannabinol; TNF-α—tumor necrosis factor-alpha; TRPV1—transient receptor potential vanilloid 1; UHPLC-TOF-MS—ultra-high-performance liquid chromatography time-of-flight mass spectrometry.

### 4.3. Effects of Phytocannabinoids in Other Neurodegenerative Conditions

Beyond AD and PD, phytocannabinoids have been explored for their potential therapeutic effects in other neurodegenerative disorders, notably Huntington’s disease (HD) and amyotrophic lateral sclerosis (ALS).

HD is one of the most devastating neurodegenerative disorders, marked by progressive motor dysfunction, cognitive decline, and a range of neuropsychiatric symptoms [131]. HD is clinically marked by abnormal involuntary movements (chorea), gait disturbances, cognitive decline, and psychiatric symptoms, including depression and anxiety [132]. HD is caused by an abnormal increase in the number of cytosine–adenine–guanine (CAG) repeats in the first exon of the HTT gene, located on the short arm of chromosome 4 (4p16.3). This mutation leads to the production of a mutant form of the huntingtin protein, which is specific to the disease [133].

Studies on post-mortem brain tissue from HD patients have shown a significant decline in CB_1_ receptor expression in multiple regions of the basal ganglia—including the caudate nucleus, putamen, globus pallidus, and substantia nigra—as the disease advances. These results point to a possible involvement of the ECS in the pathogenesis and progression of neurodegeneration in HD [73]. Preclinical studies have further supported this hypothesis, demonstrating decreased CB_1_ mRNA levels in the lateral striatum and in neurons isolated from the cortex and hippocampus of transgenic HD mouse models [134]. In addition, significant reductions in the CB_1_ receptor ligand binding have been observed in multiple basal ganglia regions [135]. The progressive decline in CB_1_ mRNA levels in R6/1 transgenic mice, as compared to wild type controls, appears to result from reduced transcription, reinforcing the theory that mutant huntingtin disrupts transcription factor activity [136]. Moreover, genetic depletion of the CB_1_ receptors in R6/2 transgenic mice led to pronounced motor impairment, striatal atrophy, and increased accumulation of huntingtin aggregates [75]. By contrast, the CB_2_ receptors—primarily expressed in microglia—appear to exert a protective effect against excitotoxicity in HD. Genetic ablation of the CB_2_ receptors in R6/2 mice, which express the human mutant HTT exon 1, resulted in heightened microglial activation, exacerbated symptom severity, and reduced life expectancy [137].

Using two different mouse models of HD, Valdeolivas et al. demonstrated that several altered physiological and behavioral parameters could be normalized through the neuroprotective effects of CBG. In 16-week-old male C57BL/6 mice treated with 3-nitropropionate (3-NP), CBG significantly improved motor impairments, as indicated by reduced hindlimb clasping, dystonia, and general locomotor dysfunction. Additionally, CBG exerted significant protective effects against 3-NP-induced inflammation and oxidative stress, as evidenced by the modulation of pro-inflammatory markers (COX-2, iNOS, TNF-α, IL-6) and oxidative stress markers (catalase, SOD, and GSH). However, in R6/2 transgenic mice, the protective effect of CBG was more modest, leading to the suggestion that CBG should be co-administered with another phytocannabinoid targeting the CB_1_ and/or CB_2_ receptors to enhance therapeutic efficacy [138].

In another model of HD-specific neurodegeneration induced by intrastriatal injection of quinolinic acid, the CBG derivative VCE-003.2 demonstrated protective effects by improving motor performance in the rotarod test, although it did not reduce cerebral edema. At the cellular level, this compound prevented neuronal loss, reduced microglial activation, and attenuated reactive astrogliosis [139]. Similarly, Aguareles et al. evaluated VCE-003.2 in a model of HD induced in C57BL/6 mice via bilateral intrastriatal administration of an adeno-associated virus (AAV). Treatment with the cannabinoid derivative improved motor coordination, reduced microglial activation, and lessened neurodegeneration [140].

Another phytocannabinoid, Δ^9^-THCA, also demonstrated significant neuroprotective effects in the 3-NP-induced HD model [141]. To enhance efficacy through combination therapy, Sagredo et al. examined the effects of THC and CBD, alone and in combination, in rats with 3-NP-induced HD. Cannabis botanical extracts enriched with varying proportions of THC and CBD significantly attenuated neuronal GABA deficiency and 3-NP-induced neuronal loss. Moreover, these combinations counteracted the downregulation of CB_1_ receptor expression and the upregulation of calpain, while restoring the expression of superoxide dismutase-1 (SOD1) [142].

Similarly, phytocannabinoids have shown beneficial effects in ALS, a neurodegenerative disease marked by the progressive loss of both upper and lower motor neurons. Damage to upper motor neurons results in spasticity and hyperexcitability, whereas degeneration of lower motor neurons leads to muscle weakness, fasciculations, and atrophy, ultimately culminating in progressive paralysis. Compared to other neurodegenerative conditions, ALS progresses more rapidly, with most patients experiencing fatal outcomes within 2 to 5 years from the onset of symptoms [143]. Currently, there is no curative treatment available. Riluzole remains the only approved drug with a relatively modest effect, capable of slightly delaying disease progression for certain patients [144].

Although the exact etiology remains unclear, several genetic mutations have been implicated in disease development. Among the most significant are mutations in the superoxide dismutase 1 (SOD1) gene [145] and hexanucleotide repeat expansions in the C9ORF72 gene [146]. Additionally, more than 20 other genetic mutations have been associated with ALS pathogenesis [147]. However, the presence of these mutant proteins alone does not fully account for the rapid progression and complex clinical manifestations of the disease. Increasing evidence suggests that non-neuronal cells, such as microglia, astrocytes, and oligodendrocytes, contribute to disease mechanisms. This is supported by the presence of extensive gliosis in the motor cortex of ALS patients [144,147]. Other pathological features include impaired glutamate reuptake, cytoskeletal abnormalities in motor axons and their distal terminals, as well as synaptic dysfunction. These alterations promote excitotoxicity, oxidative stress, and inflammatory responses, ultimately leading to neuronal apoptosis [148].

Excitotoxicity resulting from overstimulation of glutamate receptors is recognized as a key pathogenic mechanism contributing to neuronal degeneration in ALS. Excessive activation of kainate (KA) and AMPA receptors leads to increased intracellular calcium influx, which in turn triggers a cascade involving the activation of calpains, endonucleases, and phospholipases, ultimately culminating in neuronal death [149,150]. The endogenous cannabinoid system appears to serve as a neuroprotective mechanism in response to excitotoxic stress. This is supported by evidence showing that the synthesis of cannabinoids is upregulated under excitotoxic conditions. For instance, following the administration of excitotoxins in murine brain models, significantly elevated levels of the endocannabinoids AEA and 2-AG have been observed [151,152]. This adaptive response may form the basis for the potential therapeutic application of phytocannabinoids for ALS.

In a preclinical study using the SOD1^G93A transgenic mouse model of ALS, Bilsland et al. investigated the neuroprotective potential of the synthetic cannabinoid agonist WIN55,212-2. As disease symptoms progressed, the researchers observed a significant increase in the endogenous cannabinoids AEA and 2-AG, suggesting a possible compensatory protective response. Pharmacological intervention with WIN55,212-2 resulted in marked improvements in muscle strength, enhanced survival of motor units, and reduced muscle fatigue compared to untreated SOD1 mice. However, despite these functional benefits, the treatment did not extend long-term motor neuron survival or overall lifespan in the ALS model [153].

Using the same SOD1^G93A transgenic mouse model of ALS, Shoemaker et al. investigated the effects of two cannabinoid compounds: the non-selective CB_1_/CB_2_ receptor agonist WIN55,212-2 and the selective CB_2_ agonist AM-1241. While WIN55,212-2 produced a modest increase in survival, AM-1241 led to a significantly greater extension of lifespan, potentially linked to the increased expression of the CB_2_ receptors on microglial cells observed in ALS. Based on these findings, the authors proposed that selective CB_2_ agonists may offer greater therapeutic potential for ALS [154]. However, earlier studies had produced conflicting results regarding the efficacy of AM-1241. For instance, Kim et al. reported that, although AM-1241 delayed disease progression, it did not significantly prolong survival in SOD1^G93A mice [155].

Other cannabinoids have shown similar profiles. Cannabinol, for example, delayed the onset of hindlimb tremor by 17 days, but had no significant effect on survival. The authors suggested that this may be due to cannabinol’s antispastic effects, mediated through CB_1_ receptor activation, which could mask early symptoms without altering the underlying course of the disease, thus positioning it more as a symptomatic treatment than a disease-modifying agent [156].

THC has also been tested in the SOD1^G93A model. It was administered at lower doses (5 and 10 mg/kg body weight) before symptom onset, and at a higher dose (20 mg/kg) after symptoms appeared. Rotarod performance tests showed no significant motor improvement across any dosage groups when compared to controls. Nonetheless, modest improvements in disease progression were observed: the 10 mg/kg dose yielded a 3% increase in motor performance endurance, while the 20 mg/kg dose led to a 6% improvement. Correspondingly, survival increased by an average of 4.9 and 6.4 days, respectively. The authors emphasized the low toxicity of THC as a valuable trait, especially considering the chronic nature of ALS and the need for long-term therapies [157].

Moreno-Martet et al. investigated the effects of a combination of botanical extracts enriched with either THC or CBD, administered to SOD1^G93A transgenic mice from symptom onset through to the terminal stage of the disease. Their findings indicated a delay in disease progression, particularly during the early phases, with effects more pronounced in female mice. There was also a tendency toward increased survival in females, and in males, a protective effect against the characteristic weight loss seen in transgenic models. The study concluded that this phytocannabinoid-based formulation could represent a novel disease-modifying therapeutic option for ALS [158].

A quinone derivative of CBG, VGE-003.2, was evaluated in the SOD1^G93A transgenic mouse model of ALS. A battery of pharmacological tests was employed to assess neurological decline. The results indicated that the monitored parameters were only partially normalized or attenuated, and this occurred primarily during specific time points, particularly in the early stages of symptom onset. However, a more notable finding was the preservation of intact spinal motor neurons, an effect that appeared to correlate with a reduction in astroglial reactivity—an important feature in the progression of ALS. Complementary in vitro experiments conducted as part of the study suggested that the neuroprotective effects of VGE-003.2 may involve activation of PPAR-γ [159].

It is important to note, however, that much of the preclinical data available for HD and ALS is not very recent, with many studies dating back several years. This highlights the necessity for updated research efforts to validate and expand upon the earlier findings, particularly in light of the rapidly evolving understanding of cannabinoid pharmacology.

Overall, although data are promising, further preclinical and clinical investigations are essential to confirm the therapeutic potential of phytocannabinoids in these and other neurodegenerative conditions.

## 5. Conclusions

Recent advances in cannabinoid research have shed light on the considerable therapeutic potential of phytocannabinoids, particularly CBD, in the treatment of neurodegenerative diseases. The preclinical studies presented in this review demonstrate consistent neuroprotective, anti-inflammatory, antioxidant, and neuromodulator effects in models of AD, PD, or HD. These effects are largely mediated through the complex interplay of phytocannabinoids with the ECS, as well as their interactions with non-cannabinoid targets, such as TRPV1, 5-HT1A receptors, and PPARs.

The ECS emerges as a crucial modulator of CNS homeostasis, and its dysregulation appears to be closely linked with the pathophysiology of major neurodegenerative diseases. Phytocannabinoid-mediated modulation of ECS activity has shown promising outcomes in various animal models, including reductions in neuroinflammation, attenuation of excitotoxicity, and preservation of cognitive and motor function.

The evidence suggests that phytocannabinoids may contribute to neuronal preservation, attenuation of neuroinflammatory cascades, and improvement in motor and cognitive performance in disease models. Moreover, their favorable safety profile and ability to act on multiple molecular pathways position them as promising candidates for disease-modifying interventions.

Despite these advances, the translation of preclinical findings into clinical applications remains challenging. Limitations such as variability in cannabinoid content, differences in pharmacokinetics across formulations, and the lack of standardized dosing regimens underscore the need for further investigation. Moreover, the limited number of quality clinical trials hinders definitive conclusions regarding efficacy and safety in human populations.

Future research should focus on the optimization of cannabinoid-based formulations, exploration of synergistic effects among phytocannabinoids, and rigorous clinical evaluation in neurodegenerative disease populations. As interest in cannabinoid pharmacotherapy continues to grow, phytocannabinoids represent a promising, multifaceted class of compounds with the potential to address unmet therapeutic needs in the field of neurodegeneration.

## Figures and Tables

**Table 1:** Preclinical studies evaluating the effects of phytocannabinoids in AD.

Animal Model	Treatment	Results	Reference
APPS/PS1ΔE9 transgenic mice	CBD 5 mg/kg, i.p. 3 weeks	Reversed object recognition deficits. Improved spatial learning.	[104]
TAU58/2 transgenic mice	CBD 100 mg/kg bw, i.p. 3 weeks	Restored spatial reference memory. Reduced anxiety-like behaviors. Restored impaired motor function. Decreased contextual fear-associated freezing.	[105]
APP/PS1 transgenic mice	CBD 5 mg/kg bw, i.p. 30 days	Enhanced the immune system response. Upregulated autophagy in the hippocampus. Reduced Aβ plaques.	[106]
AβPPxPS1 transgenic mice	CBD 50 mg/kg bw, i.p. 3 weeks	Restored social recognition memory. Reversed spatial learning deficits. Moderately reduced insoluble Aβ_40_ levels in the hippocampus.	[107]
Aβ transgenic TAU58/2 mice	CBD 50 mg/kg bw, i.p. 3 weeks	4 months old AD transgenic mice maintained normal sociability and intact social recognition memory. CBD treatment did not alter the behavioral deficits observed in transgenic males (reduced body weight, heightened anxiety, and impaired motor functions).	[108]
STZ-induced AD Wistar rats	CBD 20 mg/kg bw, i.p. 7 days	Reduced brain glucose hypometabolism memory impairment. Prevented total weight loss.	[109]
5xFAD mice	CBD: 0.273 mg/kg bw or THC: 0.205 mg/kg bw or CBD 0.273 mg/kg bw: THC 0.205 mg/kg bw i.p. 28 days	THC: Induced anxiogenic and depressant-like behaviors. CBD–THC: Improved spatial memory. All Cannabinoid Treatments: Increased cortical levels of insoluble A_β1–42_.	[110]
AlCl_3_-induced AD Wistar rats	THC–CBD 2:1 (oily extract) 50 μL, 100 μL, and 150 μL, orally 60 days	50 μL: Moderate reduction in neuronal death in the hippocampus (~50%). Slight decrease in amyloid accumulation. Small improvement in SOD activity and reduction in NO levels. 100 μL: Significant reduction in neuronal death (~70%). Noticeable decrease in Aβ levels in brain tissue. SOD activity increased by ~15%. NO levels decreased. Less amyloid plaque formation observed in brain section. 150 μL: Most effective in protecting neurons (~80%). Strongest reduction in amyloid deposition. Best improvement in SOD activity (~20% increase). Significant reduction in NO levels. Least amyloid plaque formation, with better-preserved brain tissue.	[111]
Scopolamine-provoked memory impairment Swiss mice	CBD: 1, 5, 30 mg/kg bw, i.p. or RIV: 0.5, 1, 2.5 mg/kg bw, i.p. or CBD 1 mg/kg bw, i.p. + RIV 0.5 mg/kg bw, i.p. single treatment	CBD:1 mg/kg/RIV:0.5 mg/kg: Significantly improved memory in all stages (acquisition, consolidation, and retrieval). Combination: Demonstrated a stronger memory-enhancing effect than either drug alone.	[112]
A_β1–42_-treated ICR mice	CBDA: 6 μM or THCA: 12 μM intrahippocampal injection days 3 and 4 after A_β1–42_ injection	CBDA and THCA: Reduced hippocampal Aβ and p-tau levels. Improved cognitive function. Exhibited neuroprotective effects. Alleviated calcium dyshomeostasis. Protected primary neurons.	[113]
Transgenic *C. elegans* strain	CBD 100 μM 24 h	Enhanced the glyoxalase pathway. Preventing methylglyoxal-induced cellular damage in cerebellar neurons. Increased lifespan and survival.	[114]
Transgenic *C. elegans* expressing A_β1–42_	CBD 100 μM	Reduced Aβ aggregation. Ameliorated Aβ-associated neurotoxicity, while scavenging ROS) through CBD’s intrinsic antioxidative properties.	[115]
Transgenic *C. elegans* strain GRU102	CBD 5μM	Extended lifespan. Improved pumping rate. Reduced mitochondrial oxidative stress.	[116]

Aβ—amyloid-beta; Aβ_1–42—_amyloid-beta 1–42 peptide; Aβ_40_—amyloid-beta 40 peptide; AD—Alzheimer’s disease; AlCl_3_—aluminum chloride; bw—body weight; CBD—cannabidiol; CBDA—cannabidiolic acid; i.p.—intraperitoneal; NO—nitric oxide; p-tau—phosphorylated tau protein; RIV—rivastigmine; ROS—reactive oxygen species; SOD—superoxide dismutase; STZ—streptozotocin; THCA—tetrahydrocannabinolic acid.

## Data Availability

No new data were created or analyzed in this study.
